# Efficacy and Safety of Recombinant Human Prourokinase in Acute Ischemic Stroke: A Systematic Review and Meta-Analysis of Randomized Controlled Trials

**DOI:** 10.3390/brainsci15050466

**Published:** 2025-04-28

**Authors:** Haneen Sabet, Abdallah Abbas, Mohamed El-Moslemani, Mohamed Ahmed Zanaty, Ramanathan Kadirvel, Sherief Ghozy

**Affiliations:** 1 Faculty of Medicine, South Valley University, Qena 1453055, Egypt; haninsabet225@gmail.com (H.S.); mohamed.ahmed.zanaty24@gmail.com (M.A.Z.); 2 Faculty of Medicine, Al-Azhar University, Damietta 7991164, Egypt; abdallah.abdelmoneam.abbas@gmail.com (A.A.); drmohamedelmeslimany@gmail.com (M.E.-M.); 3 Department of Neurologic Surgery, Mayo Clinic, Rochester, MN 55902, USA; kadir@mayo.edu; 4 Department of Radiology, Mayo Clinic, Rochester, MN 55902, USA

**Keywords:** acute ischemic stroke, recombinant human prourokinase (rhPro-UK), intravenous thrombolysis, intra-arterial thrombolysis, alteplase

## Abstract

**Objective:** To evaluate the safety and efficacy of recombinant human prourokinase (rhPro-UK) administered via intravenous (IV) and intra-arterial (IA) routes in acute ischemic stroke (AIS) patients compared with standard treatments. **Methods:** A comprehensive search was conducted in accordance with PRISMA guidelines across Scopus, Web of Science, and PubMed until 11 December 2024. Randomized controlled trials (RCTs) assessing rhPro-UK’s efficacy and safety were included. Outcomes included the modified Rankin Scale (mRS), the National Institutes of Health Stroke Scale (NIHSS), mortality, and adverse events (AEs). Data analysis used risk difference (RD) with 95% confidence intervals (CIs). **Results:** Six RCTs (n = 3993) met the inclusion criteria. IV rhPro-UK showed comparable efficacy to the comparator for the mRS 0–1 at 90 days (RD: 0.00, 95% CI: [−0.04, 0.04]) and the mRS 0–2 (RD: −0.01, 95% CI: [−0.03, 0.01], P = 0.23). IA rhPro-UK significantly improved the mRS 0–1 (RD: 0.13, 95% CI: [0.01, 0.26], P = 0.04). The NIHSS reduction was significant for IV rhPro-UK (MD: −0.83, 95% [CI: −1.36, −0.29]). IV rhPro-UK did not significantly reduce the risk of systemic bleeding (RD: −0.10, 95% CI: [−0.24, 0.03], P = 0.12), serious AEs (RD: −0.01, 95% CI: [−0.04, 0.02], P = 0.53), or mortality (RD: 0.01, 95% CI: −0.01, 0.02). IA rhPro-UK significantly increased hemorrhage with neurological deterioration (RD: 0.08, 95% CI: [0.01, 0.14], P = 0.02). **Conclusions:** IV rhPro-UK provides non-inferior efficacy to both alteplase and standard care with a better safety profile at 35 mg, while IA rhPro-UK enhances functional outcomes in middle cerebral artery occlusions, albeit with safety concerns. Further trials are needed to confirm long-term outcomes, optimal dosing, and broader applicability.

## 1. Introduction

As one of the leading causes of morbidity and mortality worldwide, acute ischemic stroke requires timely and effective treatment strategies [1]. Current guidelines recommend intravenous (IV) thrombolysis with a recombinant tissue plasminogen activator (rt-PA, alteplase) as the first-line treatment for eligible AIS patients within a narrow therapeutic window of 4.5 h from the onset of symptoms [1]. However, limitations associated with alteplase, including systemic bleeding risks, cost, and logistical challenges, have spurred interest in alternative thrombolytic agents [2,3,4].

Recombinant human prourokinase (rhPro-UK), a plasminogen activator with fibrin specificity, has emerged as a promising alternative [2]. Its unique pharmacodynamic properties minimize systemic plasminogen activation, thereby reducing the risks of systemic bleeding and intracranial hemorrhage (ICH) observed with alteplase [2]. Earlier findings have demonstrated the efficacy and safety of rhPro-UK administered via IV and intra-arterial (IA) routes [1,2,3,4,5,6]. The PROACT trials were pivotal in highlighting the potential of IA prourokinase in achieving significant recanalization and favorable functional outcomes in patients with AIS caused by middle cerebral artery (MCA) occlusions [5,6].

Recent phase II and III trials have evaluated IV rhPro-UK in direct comparison with alteplase, showing non-inferior efficacy in achieving positive outcomes, as assessed by modified Rankin Scale (mRS) scores, with reduced risks of systemic bleeding [2,3]. The PROST trials further emphasized its potential as a cost-effective and accessible alternative in resource-limited settings [2,3,4].

Despite these promising results, comprehensive evaluations of the comparative efficacy and safety of IV and IA rhPro-UK in AIS management remain limited. This review and meta-analysis has the objective of evaluating the efficacy and safety of rPro-UK administered via IV and IA routes in AIS patients according to evidence from randomized controlled trials (RCTs). By pooling data from available studies, we seek to provide robust insights into the clinical utility of rhPro-UK, thus informing practice and guiding future research.

## 2. Methods

We worked on this systematic review and meta-analysis following the Preferred Reporting Items for Systematic Reviews and Meta-Analyses (PRISMA) and Cochrane Guide for Systematic Reviews of Interventions [7,8].

### 2.1. Search and Eligibility Criteria

We searched Scopus, Web of Science, and PubMed from their inception to 11 December 2024, applying the search strategy: ((Prourokinase OR Pro-urokinase OR “Recombinant Prourokinase” OR “recombinant human prourokinase” OR “rhPro-UK” OR “rpro-UK”) AND (Stroke OR “Ischemic stroke” OR “Acute ischemic stroke” OR “AIS” OR “Cerebral infarction” OR “Acute cerebral ischemia” OR “Cerebral ischemia” OR “Embolic stroke” OR “Thrombotic stroke” OR “Ischemic brain injury”)).

All RCTs that assessed the safety, efficacy, or both of IV rhPro-UK or IA rhPro-UK in AIS patients were included. Trials were included regardless of the comparator treatment (e.g., alteplase, heparin with or without saline) to allow for comprehensive analyses. Studies were eligible for inclusion regardless of language. To ensure high-quality evidence, only RCTs were considered, and we excluded other study designs such as case–control studies, cohort studies, cross-sectional studies, editorials, abstracts, conference proceedings, and studies involving different interventions. Only fully published studies were included to ensure complete data availability.

### 2.2. Screening and Data Extraction

We screened articles utilizing Rayyan software [9] in two steps: first by reviewing titles and abstracts, and then by reviewing full texts. Two independent authors carried this out, referring any disagreements to the first author.

Two independent authors extracted data using Excel and referred any disagreements to the first author. Data extraction involved the following:

(a) Data summary: Study ID, country, duration, study design, population, intervention, comparator, route of administration of rhPro-UK, outcomes measured, summary of the study.
(b) Baseline data: Sample size, age, gender, body weight, systolic and diastolic blood pressure.
(c) Outcomes: efficacy outcomes (mRS scores, National Institutes of Health Stroke Scale (NIHSS) scores) and safety outcomes (mortality, symptomatic ICH, ICH, systemic bleeding, serious adverse events (AEs)). We extracted all the prespecified safety and efficacy outcomes that were reported in at least two of the included RCTs.

### 2.3. Bias Risk Assessment

The risk of bias assessment was conducted independently by two authors using the RoB-2 tool, used to assess the quality of RCTs [10].

### 2.4. Data Analysis

We conducted data analysis using RevMan 5.4 software [11] and utilized the Meta-Analysis Accelerator tool to perform statistical conversions [12]. For continuous data, we analyzed the pooled mean difference (MD) with 95% confidence intervals (CIs). For categorical data, we calculated the risk difference (RD) with 95% CIs. We specifically have chosen the RD because it is one of the effect sizes that enables us to calculate the number needed to treat (NNT) [13], which is clinically meaningful and derived from the RD. Calculating the NNT using RD has been previously described in the literature [14,15,16].

In the context of efficacy outcomes, the NNT represents the average number of patients who need to be treated for one additional patient to benefit from the intervention compared to the control group. For safety outcomes, such as AEs, the NNT becomes the number needed to harm (NNH), indicating the average number of patients treated for one additional patient to experience a specific AE. Lower NNH values suggest a higher risk associated with the intervention, which is crucial for assessing the risk–benefit balance.

Our analysis approach included a comprehensive evaluation of the data through subgroup analyses and pooled overall effect sizes. Specifically, we performed subgroup analyses for IV rhPro-UK and IA rhPro-UK, allowing for a detailed comparison of the efficacy and safety profiles of each administration route. Additionally, we conducted subgroup analyses based on the dose of IV rhPro-UK, enabling us to assess the impact of different doses on clinical outcomes. These subgroup analyses were complemented by the calculation of pooled overall effect sizes to provide an integrated summary of treatment effects across all included studies.

We assessed heterogeneity using the chi-square test and I-squared (I²) statistics. Heterogeneity was considered significant when P < 0.1 or I² > 50% [17]. A random-effects model was utilized due to the suspected heterogeneity and variability among studies [18]. We did not assess publication bias, as this requires a minimum of ten studies, per Cochrane guidelines [19].

## 3. Results

### 3.1. Search and Screening

**Figure 1:**
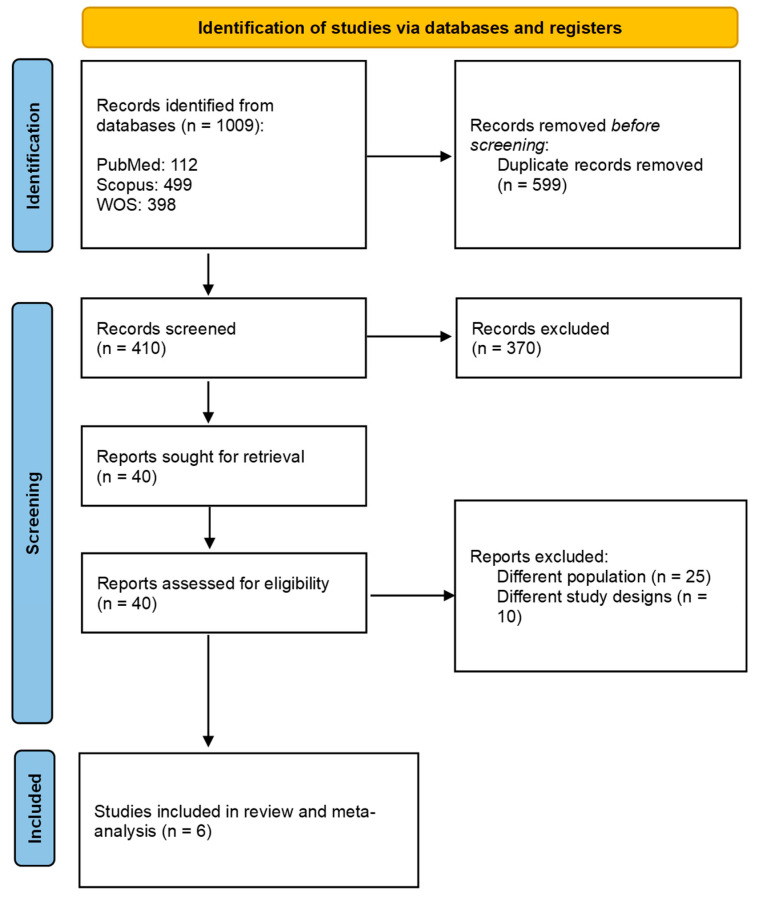
rhPro-UK group vs. comparator group. PRISMA flowchart of the search and selection process.

A total of 1009 articles were identified from the searched databases. After removing duplicates, the number of articles was reduced to 410. Following the screening of titles and abstracts, the selection was narrowed down to 40 articles, excluding any studies that did not meet our previously mentioned inclusion criteria. Ultimately, six studies were included after the full-text screening: three focused on IV rhPro-UK [2,3,4,20] and two on IA rhPro-UK [5,6] (see Figure 1).

### 3.2. Summary and Baseline

The total sample size across all studies was 2049 participants in the rhPro-UK group and 1944 participants in the control group. The gender distribution showed that the rhPro-UK group included 891 males and 435 females, while the control group included 1363 males and 624 females. The pooled mean age in the rhPro-UK group was 64.24 years, with a pooled standard deviation (SD) of 11.42 years, compared to a pooled mean age of 64.07 years and an SD of 10.67 years in the control group. Additional variables such as body weight; systolic and diastolic blood pressure; and outcomes such as the mRS, the NIHSS, the Barthel Index, safety measures, and recanalization rates are detailed in Table 1 and Table 2. These tables provide further insights into the baseline characteristics and outcomes of the included studies.

### 3.3. Risk of Bias Assessment

A total of four studies [2,3,4,20] on IV rhoPro-UK were evaluated; all were rated as having “some concerns”. All domains were assessed as having a low risk of bias, except for Domain 2, which was marked with “some concerns” due to the open-label study design that could potentially influence the findings. Among the other two studies, one [5] was also rated as having “some concerns”, while the other [6] was assessed as having a “high risk” of bias (see Supplementary Figure S1).

### 3.4. IV rhPro-UK vs. Alteplase Analysis

#### 3.4.1. Analysis of Efficacy Outcomes

**Figure 2:**
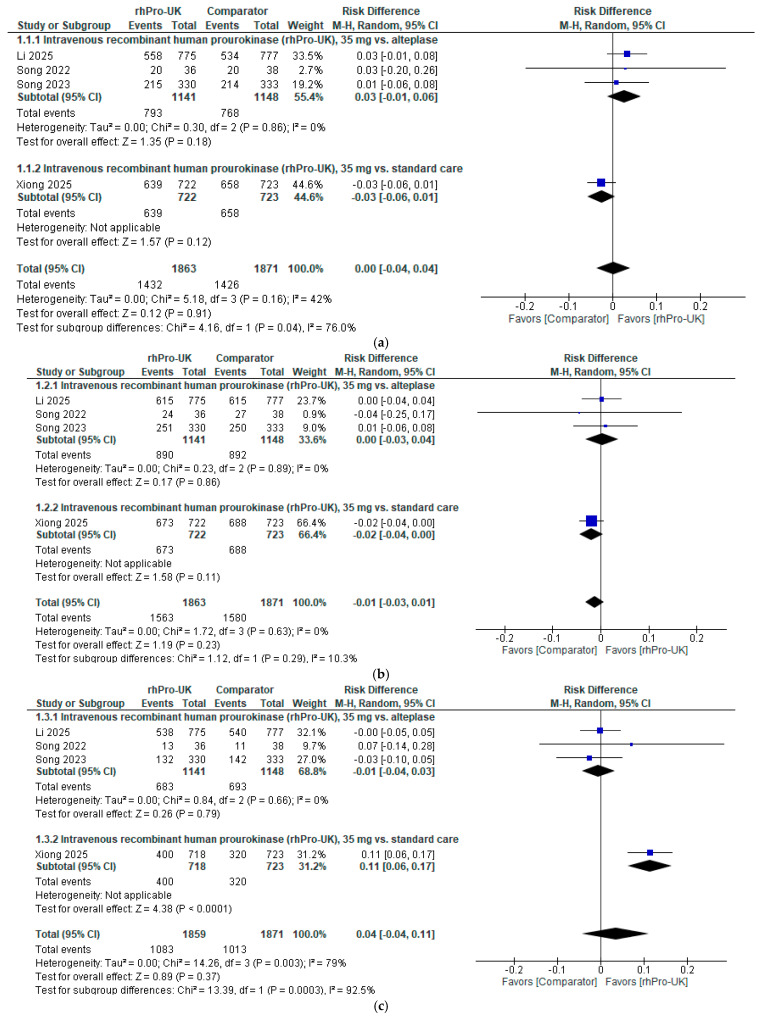

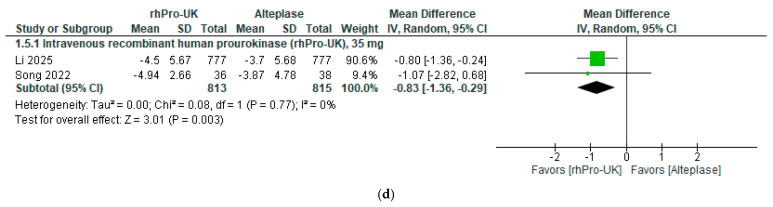
IV rhPro-UK group vs. alteplase group [2,3,4,20]: (**a**) 90-day mRS (0–1 rates), (**b**) 90-day mRS (0–2) rates, (**c**) NIHSS (0–1) rates at the end of follow-up, (**d**) NIHSS decrease at the end of follow-up.

Regarding the 90-day mRS (0–1), the overall analysis of IV rhPro-UK (35 mg) showed no significant difference compared to the control group (RD: 0.00, 95% CI: [−0.04, 0.04], P = 0.91). The RD is below the minimal clinically important difference (MCID) threshold (NNT: 4 to 13) [21], and the CI includes zero, indicating that there is no clinically meaningful difference. The data showed no heterogeneity (I² = 42%, P = 0.16). Furthermore, a subgroup analysis based on the control group, either alteplase or standard care (anticoagulant or dual antiplatelet therapy), demonstrated no significant differences between IV rhPro-UK and the comparator in any subgroup (refer to Figure 2a).

Regarding the 90-day mRS (0–2), the overall analysis of IV rhPro-UK (35 mg) showed no significant difference compared to the control group (RD: −0.01, 95% CI: [−0.03, 0.01], P = 0.23). The RD is below the MCID threshold [21], and the CI includes zero, indicating that there is no clinically meaningful difference. The data showed no heterogeneity (I² = 0%, P = 0.63). Furthermore, a subgroup analysis stratified by control group, either alteplase or standard care, demonstrated no significant differences between IV rhPro-UK and the comparator in any subgroup (refer to Figure 2b).

Regarding the NIHSS score 0–1, the overall analysis of IV rhPro-UK (35 mg) showed no significant difference compared to the control group (RD: 0.04, 95% CI: [−0.04, 0.11], P = 0.37). The RD is below the MCID threshold [21], and the CI includes zero, indicating that there is no clinically meaningful difference. There was significant heterogeneity in the overall analysis (I² = 0.79%, P = 0.003). After excluding the Xiong 2025 [20] study, heterogeneity was resolved (I² = 0%, P = 0.66) (see Supplementary Figure S2). Furthermore, a subgroup analysis was conducted based on the control group: when compared to alteplase, there was no significant difference; however, when compared to standard care, there was a significant difference (RD: 0.11; 95% CI: [0.06,0.17]; P < 0.0001) (refer to Figure 2c).

Regarding the NIHSS (mean difference), a significant reduction in the NIHSS scores was observed for the 35 mg dose of IV rhPro-UK compared to alteplase (MD: −0.83, 95% CI: [−1.36, −0.29], *p* = 0.003). The data showed no heterogeneity (I^2^ = 0%, *p* = 0.77) (refer to Figure 2d).

#### 3.4.2. Analysis of Safety Outcomes

**Figure 3:**
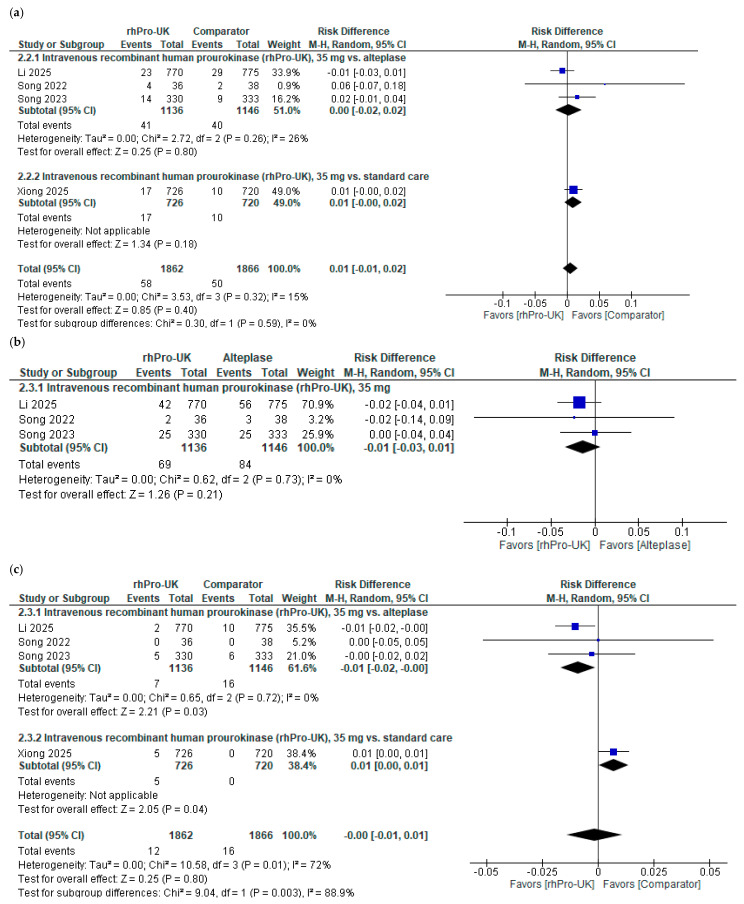

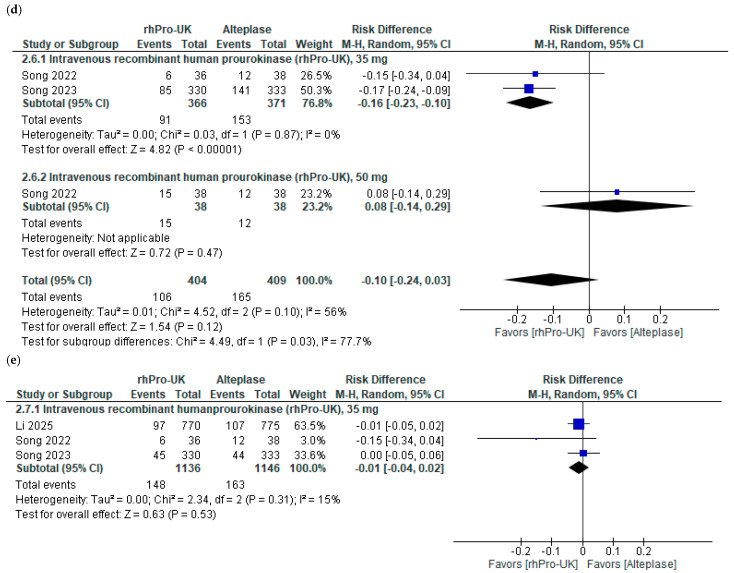
IV rhPro-UK group vs. comparator group [2,3,4,20]: (**a**) mortality risk, (**b**) ICH risk, (**c**) symptomatic ICH risk, (**d**) systemic bleeding at 90 days risk, (**e**) serious AE risk.

Regarding 90-day mortality, the overall analysis of IV rhPro-UK (35 mg) showed no significant difference compared to the control group (RD: 0.01, 95% CI: [−0.01, 0.02], P = 0.4). The RD equals 0.01, which is below the MCID threshold [21]; moreover, the CI crosses zero, indicating no clinically meaningful difference. The data were homogenous (I^2^ = 26%, *p* = 0.26). Furthermore, a subgroup analysis stratified by the control group, either alteplase or standard care, demonstrated no significant differences between IV rhPro-UK and the comparator in any subgroup (see Figure 3a).

Regarding the ICH risk, no significant difference was observed between IV rhPro-UK (35 mg) and alteplase (RD: −0.01, 95% CI: [−0.03, 0.01], P = 0.21). An RD of –0.01 falls below the MCID threshold [21]; moreover, the CI crosses zero, indicating no clinically meaningful difference. The data were homogenous (I² = 0%, P = 0.73) (see Figure 3b).

Regarding the sICH risk, compared to the control group, the overall analysis of IV rhPro-UK revealed no significant reduction in the risk of sICH compared to the control group (RD: −0.00, 95% CI: [−0.01, 0.01], P = 0.8). The RD falls below the MCID threshold [21]; moreover, the CI crosses zero, indicating no clinically meaningful difference. There was significant heterogeneity in the overall analysis (I² = 0.72%, P = 0.01). After excluding the Xiong 2025 [20] study, heterogeneity was resolved (I² = 0%, P = 0.72) (see Supplementary Figure S3). Furthermore, a subgroup analysis was conducted based on the control group: when compared to alteplase, there was no significant difference; however, when compared to standard care, there was a significant difference (RD: 0.01; 95% CI: [0.00,0.01]; P = 0.04) (see Figure 3c).

Regarding the 90-day systematic bleeding risk, the overall analysis of IV rhPro-UK revealed no significant reduction in the risk of systemic bleeding at 90 days compared to alteplase (RD: −0.10, 95% CI: [−0.24, 0.03], *p* = 0.12) (see Figure 3d). The studies showed moderate heterogeneity (I^2^ = 56%, *p* = 0.10). In a subgroup analysis of different doses of IV rhPro-UK, a significant reduction in the risk of systemic bleeding was observed with 35 mg of IV rhPro-UK compared to with alteplase (RD: −0.16, 95% CI: [−0.23, −0.10], *p* < 0.00001). The data were homogenous (I^2^ = 0%, *p* = 0.87). The RD of −0.16 corresponds to an NNT of approximately six, suggesting that treating six patients with 35 mg of IV rhPro-UK instead of alteplase prevents one case of systemic bleeding. This falls within the MCID range (NNT: 4 to 13) [21], and with the CI not including zero, it indicates a clinically meaningful difference. However, the risk of systemic bleeding did not differ significantly between 50 mg of IV rhPro-UK and alteplase (RD: 0.08, 95% CI: [−0.14, 0.29], P = 0.47).

Regarding the risk of serious AEs, no significant difference was observed for 35 mg of IV rhPro-UK compared to for alteplase (RD: −0.01, 95% CI: [−0.04, 0.02], P = 0.53). A risk difference of –0.01 falls below the MCID threshold [21]; moreover, the confidence interval crosses zero, indicating no clinically meaningful difference. Data were homogenous (I² = 15%, P = 0.31) (refer to Figure 3e).

### 3.5. IA rhPro-UK Analysis

#### 3.5.1. Analysis of Efficacy Outcomesc

**Figure 4:**
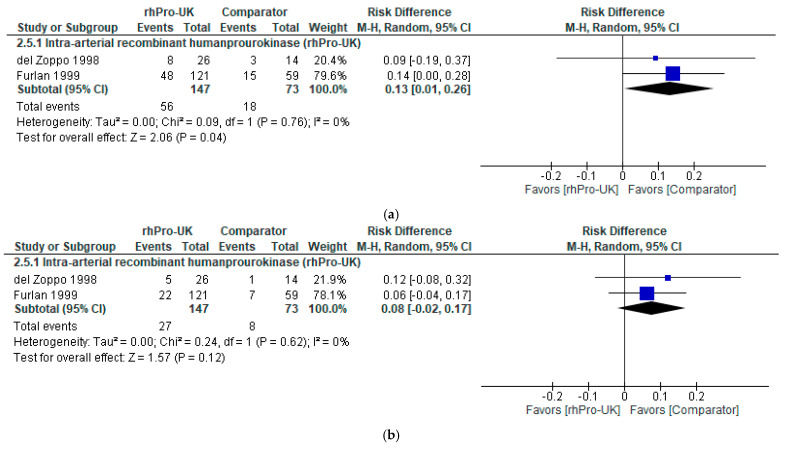
IA rhPro-UK group vs. comparator group [5,6]: (**a**) 90-day mRS (0–1) rates, (**b**) NIHSS (0–1) rates.

Regarding the 90-day mRS (0–1), IA rhPro-UK was associated with a significant increase in favorable outcomes compared to heparin in Furlan et al. [5] and heparin with saline placebo in Del Zoppo et al. [6] (RD: 0.13, 95% CI: [0.01, 0.26], P = 0.04). The RD of 0.13 corresponds to an NNT of approximately eight, within the MCID range (NNT: 4 to 13) [21], suggesting that about eight patients would need to be treated to achieve a favorable outcome (mRS 0–1) in one additional patient. With the confidence interval not including zero, this indicates that the results are both statistically and clinically significant. The data were homogenous (I² = 0%, P = 0.76) (refer to Figure 4a).

Regarding the NIHSS score 0–1, IA rhPro-UK showed no significant difference compared to heparin and heparin with saline placebo groups (RD: 0.08, 95% CI: [−0.02, 0.17], P = 0.12). The RD indicates a number needed to treat (n = 13), which falls within the MCID range (NNT: 4 to 13) [21]; however, the CI includes zero, which indicates that there is no clinically meaningful difference. The data were homogenous (I² = 0%, P = 0.62) (refer to Figure 4b).

#### 3.5.2. Analysis of Safety Outcomes

**Figure 5:**
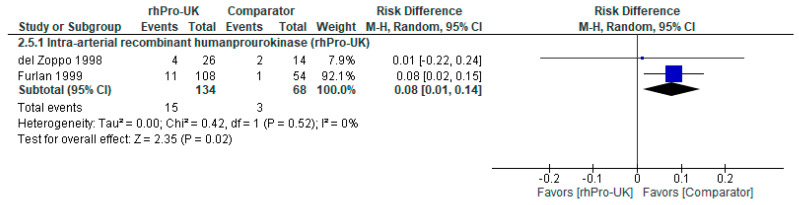
IA rhPro-UK group vs. comparator group [5,6] risk of hemorrhage with neurological deterioration.

Regarding risk of hemorrhage with neurological deterioration, the overall analysis of IA rhPro-UK favored the comparator, showing a statistically significant difference compared to heparin and saline in Del Zoppo et al. (1998) [6] and heparin in Furlan et al. (1999) [5] (RD: 0.08, 95% CI: [0.01, 0.14], P = 0.02) (see Figure 5). The data were homogenous with no heterogeneity detected (I² = 0%, P = 0.52). The RD of 0.08 corresponds to an NNH of approximately 13, meaning 13 patients treated with IA rhPro-UK would result in one additional case of hemorrhage with neurological deterioration. Although statistically significant, the RD indicates an NNH below the MCID threshold (NNH = 37) [21], and the confidence interval borders zero, suggesting the difference may not be clinically meaningful.

## 4. Discussion

Our study evaluated the efficacy and safety of rhPro-UK for AIS, comparing clinical outcomes with standard treatments (alteplase, direct oral anticoagulant, or antiplatelet therapy and heparin with or without saline placebo). IV rhPro-UK demonstrated comparable efficacy to alteplase with non-inferior results in achieving favorable clinical outcomes (mRS 0–1 or 0–2) at 90 days. IA rhPro-UK showed significant improvements in achieving favorable outcomes (mRS 0–1) compared to heparin with and without saline placebo. Both administration routes exhibited acceptable safety profiles. IV rhPro-UK significantly reduced systemic bleeding risks compared to alteplase, particularly at the 35 mg dose. However, IA rhPro-UK showed a significant risk of hemorrhage with neurological deterioration. IV rhPro-UK matched ateplase and standard care in clinical efficacy while demonstrating a reduced risk of systemic bleeding, highlighting its potential safety advantage. IA rhPro-UK improved functional outcomes (mRS 0–1) but had some concerns regarding specific safety aspects.

When given within 4.5 h after the onset of symptoms, alteplase is the most effective treatment for AIS, per the American Heart Association’s guidelines [22]. Its safety profile is questionable though; therefore, before taking alteplase, patients should be checked for contraindications, such as bleeding problems or recent surgery [22]. Other medications that might be safer and just as effective as alteplase—or even better—are the subject of more research. Our study found that the IV rhPro-UK is not inferior to alteplase or standard care, and those who took the 35 mg dose had low systematic bleeding compared to those who took alteplase.

In China, a phase 3 RCT [2] examined the efficacy and safety of IV rhPro-UK administered within 4.5 h of the onset of AIS. At 90 days, 65.2% of the rhPro-UK group patients and 64.3% of the alteplase group patients had an mRS score of 0 or 1, indicating that neither medication was worse than the other. Symptomatic ICH occurred in 1.5% of patients who received rhPro-UK and 1.8% of patients who received alteplase. This indicates that the two treatments are roughly equally safe. However, compared to the alteplase group (42.2%), the rhPro-UK group saw significantly less systemic bleeding within 90 days (25.8%). The two groups had comparable 90-day mortality rates (1.5%). Additionally, the PROST-2 trial [4] examined the safety and efficacy of IV rhPro-UK in treating patients with AIS within 4.5 h of the onset of symptoms in comparison to alteplase. IV-rhPro-UK was determined to be no worse than alteplase. Only 68.7% of the alteplase group patients had a positive prognosis (mRS 0–1) at 90 days, compared to 72.0% of patients in the rhPro-UK group (RR: 1.10, 95% CI: [0.98, 1.10]). The safety profile, however, preferred IV rhPro-UK over alteplase; within 36 h, 0.3% of the rhPro-UK group experienced symptomatic cerebral bleeding, compared to 1.3% of the alteplase group (P = 0.021). Within 7 days, 2.1% of patients receiving alteplase and 0.5% of patients receiving rhPro-UK experienced major bleeding (P = 0.0072). However, there was no difference in all-cause mortality between alteplase and IV rhPro-UK. In a nutshell, these two RCTs and our meta-analysis show that IV rhPro-UK is just as successful as alteplase in producing positive functional results, with a similar safety profile and a decreased risk of systemic bleeding.

The four included RCTs ultimately focused on the 35 mg dosage of IV rhPro-UK. However, one of these trials, Song et al. (2022) [3], also investigated a 50 mg dose. Their findings indicated that 50 mg was not inferior to alteplase in terms of efficacy and had a comparable safety profile. However, they observed that the 35 mg dosage was associated with a better safety profile, particularly with a lower incidence of serious AEs and symptomatic ICH. Based on these findings, the second RCT [2] focused exclusively on the 35 mg dose, likely due to its favorable balance between efficacy and safety, making it the preferred candidate for further investigation.

Urokinase, another thrombolytic agent used in AIS, was evaluated in the TRUST trial [23]. The study found no functional benefit of urokinase when compared to the guideline-based therapy for minor strokes (84.9% vs. 85.7%, p = 0.87); moreover, the urokinase group had a non-significantly higher rate of sICH (0.6% vs. 0.2%, p = 0.62) [23]. A meta-analysis of urokinase versus alteplase reported similar results, showing comparable efficacy (73.8% vs. 80.6%, p = 0.18) and similar sICH risk profiles (2.8% vs. 1.8%, p = 0.41) between the two groups [24]. In contrast, our findings on rhPro-UK reported non-inferior efficacy compared to alteplase, but it significantly reduced the risk of sICH (P = 0.03).

The PROACT phase II trial [6] examined the safety and efficacy of IA rhPro-UK in recanalization in patients with a confirmed MCA occlusion who received treatment within six hours of the onset of symptoms. At 120 min, the rhPro-UK group experienced a significantly higher rate of recanalization (57.7%) than the heparin with the saline placebo group (14.3%) (P = 0.017). However, compared to 7.1% of participants in the heparin with saline placebo group, 15.4% of participants in the rpro-UK group experienced symptomatic ICH within 24 h, resulting in neurological deterioration (P = 0.64). The study also found that the rate of bleeding and the rate of recanalization were influenced by the amount of heparin utilized. Higher heparin dosages have increased the risk of hemorrhagic transformation and rates of recanalization. Additionally, the PROACT II research [5] revealed that 40% of patients in the IA rhPro-UK + heparin group had a 90-day mRS 0–2, which is 15% absolute benefit and an NNT of seven, compared to 25% of patients in the heparin-only group (P = 0.04). The recanalization rate was significantly greater in the IA rhPro-UK + heparin group (66% vs. 18%) than in the heparin-only group (P < 0.001). The groups’ 90-day mortality rates were comparable (25% vs. 27%). However, compared to just 2% of those in the heparin-only group, 10% of those in the rhPro-UK + heparin group experienced ICH symptoms and neurological deterioration within 24 h (P = 0.06).

Our meta-analysis faces several limitations. Only six RCTs met the inclusion criteria, limiting the robustness of the conclusions, particularly in subgroup analyses. There are concerns about the risk of bias in the included studies, particularly the IA rhPro-UK studies, with some studies flagged for “some concerns” or “high risk” in domains like outcome measurement and reporting, which might influence the reliability of pooled results. Sensitivity analysis excluding the high-risk study was not feasible due to the limited number of studies. Therefore, the findings should be interpreted with caution. This highlights the need for more high-quality, low-risk RCTs to strengthen the evidence. Furthermore, there may be a possible patient overlap between two of the included studies [4,20], as both were conducted in China with partially overlapping recruitment periods, although neither study addressed this in their methodology. Additionally, the variations in rhPro-UK dosages, comparators, patient populations, and follow-up durations complicate direct comparisons and may introduce variability in the findings. For instance, some studies had follow-up periods extending to 90 days, while others reported outcomes for the NIHSS over shorter intervals, making it challenging to achieve uniform conclusions. Another limitation is the focus on 90-day outcomes, leaving the long-term effects of rhPro-UK unexplored. This study also does not formally assess publication bias due to the limited number of studies included, which could cause the treatment effects to be overestimated. Lastly, while the trials represent data from regions like China and the USA, the geographic concentration may restrict the external validity of the findings for broader populations.

### 4.1. Implications for Clinical Practice

IV rhPro-UK appears to be a safer alternative to alteplase, particularly for patients where systemic bleeding risk is a primary concern. This superiority was observed only with the 35 mg dose, which significantly reduced systemic bleeding, while the 50 mg dose did not differ from alteplase. IA rhPro-UK may be more effective for improving functional outcomes in specific patient populations, such as those with MCA occlusions, but its use should be carefully considered due to potential safety concerns. Ultimately, the choice of administration route should align with the patient’s clinical presentation, available expertise, and risk profile.

### 4.2. Gap of Knowledge and Future Research Recommendations

First, most studies focus on functional outcomes at 90 days. Long-term, cognitive, quality of life, and survival outcomes are underexplored. Additionally, there is limited data on how patient subgroups (e.g., those with specific comorbidities, older age, or more severe strokes) respond to rhPro-UK compared to alteplase and standard care. Moreover, current studies are predominantly based in China, raising questions about generalizability to diverse populations and healthcare systems. Finally, the potential of combining rhPro-UK with other interventions, such as mechanical thrombectomy, remains insufficiently studied. While rhPro-UK is noted to be cost-effective, comprehensive economic evaluations across different healthcare systems are scarce.

Based on these gaps, we recommend several key areas for future research. Firstly, conducting multicenter, international trials to validate findings in varied populations. Secondly, exploring combination therapies and varying administration routes to maximize outcomes. Finally, evaluating long-term and real-world data to address efficacy, safety, and cost-effectiveness comprehensively.

## 5. Conclusions

This systematic review and meta-analysis demonstrates that rhPro-UK offers a promising alternative to alteplase and standard care for AIS management. IV rhPro-UK exhibits non-inferior efficacy with a significantly reduced risk of systemic bleeding, particularly at the 35 mg dose, compared to alteplase. IA rhPro-UK demonstrates superior favorable functional outcomes in MCA occlusions, albeit with a slightly elevated risk of hemorrhage with neurological deterioration in specific settings. While these findings highlight the clinical utility of rhPro-UK, variations in dosing, administration routes, and study designs necessitate further research to solidify its safety and efficacy profile. Future investigations should explore long-term outcomes, patient subgroup responses, and its integration with mechanical thrombectomy. These efforts will help establish rhPro-UK as a cost-effective, accessible, and safe alternative in diverse healthcare systems.

## Figures and Tables

**Table 1:** Summary of included studies.

Study ID	Country	Duration	Design	Population	Intervention	Comparator	Route of Administration of rhPro-UK	Outcomes Measured	Summary of the Study
**Xiong 2025 [20]**	China	2022–2023	RCT	Patients with mild ischemic stroke (NIHSS ≤ 5).	IV rhPro-UK (15 + 20 mg)	Standard care (antiplatelet or anticoagulant therapy)	IV	mRS score ≤ 1 at 90 days safety outcomes	Prourokinase was not superior to standard care in improving functional outcomes in mild stroke but had a similar safety profile.
**Li 2025 [4]**	China	2023–2024	RCT	Patients over 18 years old with acute ischemic stroke who were ineligible for or refused endovascular thrombectomy.	IV rhPro-UK (15 + 20 mg, within 30 min)	Alteplase	IV	mRS, NIHSS, Safety	In this trial of 1555 patients, rhPro-UK was found to be non-inferior to alteplase in achieving excellent functional outcomes, with no difference in safety endpoints between the groups.
**Song 2023 [2]**	China	2018–2020	RCT	Patients aged 18 to 80 years and diagnosed with AIS who experienced stroke onset within 4.5 h and had an NIHSS stroke severity score between 4 and 25).	rhPro-UK (15 + 30 mg, within 30 min)	Alteplase	IV	mRS, NIHSS, Batheral index, safety	Of 663 patients, this trial supports that rhPro-UK is safe and that there is non-inferiority improvement in outcomes by using intravenous rhPro-UK compared to the control group.
**Song 2022 [3]**	China	2016–2017	RCT	Acute ischemic stroke patients.	Group 1: 50 mg of rhPro-UK and Group 2: 35 mg of rhPro-UK	Alteplase	IV	mRS, NIHSS, safety	In this trial of 112 patients, both low- and high-dose IV rhPro-UK were shown to be safe for AIS patients within the 4.5 h treatment window, with significant improvement in outcomes using rhPro-UK 50 mg and 35 mg doses.
**Furlan 1999 [5]**	USA	1996–1998	RCT	Patients with new-onset focal neurological signs in the MCA distribution, eligible for treatment within 6 h of symptom onset, with a minimum NIHSS score of 4 and aged 18 to 85 years.	rhPro-Uk 9 mg + heparin	Heparin only	IA	mRS, NIHSS, Batheral index, recanalization, safety	Of 180 patients, this trial supports that rhPro-UK is safe and that outcomes are superior by using intra-arterial rhPro-UK compared with the control group.
**del Zoppo 1998 [6]**	USA	1994–1995	RCT	Patients with new-onset focal neurological signs in the MCA distribution, eligible for treatment within 6 h of symptom onset, a minimum NIHSS score of 4, and aged 18 to 85 years.	rhPro-UK (6 mg) over 120 min + heparin	Saline placebo + heparin	IA	mRS, NIHSS, Batheral index, recanalization, safety	Of 40 patients, this trial supports that rhPro-UK is safe and there is a significant improvement in outcomes by using intra-arterial rhPro-UK.

**Abbreviations: RCT**: randomized controlled trial; **AIS**: acute ischemic stroke; **mRS**: modified Rankin Scale; **NIHSS**: National Institutes of Health Stroke Scale; **rhPro-UK**: recombinant human prourokinase; **IV**: intravenous; **IA**: intra-arterial.

**Table 2:** Baseline characteristics.

Study ID	Sample Size (No.)		Age, Years, Mean (SD)		Gender, Males No.–Females No.		Body Weight, kg, Mean (SD)		Systolic Blood Pressure (mmHg), Mean (SD)		Diastolic Blood Pressure (mmHg), Mean (SD)	
	rhPro-UK	Comparator	rhPro-UK	Comparator	rhPro-UK	Comparator	rhPro-UK	Comparator	rhPro-UK	Comparator	rhPro-UK	Comparator
**Xiong 2025 [20]**	723	723	65.57 (11.59)	65.37 (10.55)	479: 244	469: 254	N/A	N/A	N/A	N/A	N/A	N/A
**Li 202** **5 [4]**	775	777	64.6 (11.14)	64.33 (10.4)	503:272	529:248	67.50 (11.14)	67.67 (11.14)	N/A	N/A	N/A	N/A
**Song 2023 [2]**	330	333	61.44 (10.2)	60.57 (10.2)	256:74	246:87	69.59 (11.82)	70.49 (12.02)	151.46 (22.08)	150.06 (21.17)	87.35 (12.7)	86.48 (12.62)
**Song 2022 [3]**	74	38	rhPro-UK 35 mg: 60.87 (7.33), 50 mg: 57.92 (12.07)	62.76 (9.07)	rhPro-UK 35 mg: 23:13, 50 mg: 25:13	25:13	N/A	N/A	rhPro-UK 35 mg: 151.78 (18.30), 50 mg: 150.03 (19.01)	156.76 (18.83)	rhPro-UK 35 mg: 90.78 (14.26), 50 mg: 88.26 (11.55)	85.63 (11.15)
**Furlan 1999 [5]**	121	59	64 (14)	64 (14)	70:51	36:23	79 (16)	81 (19)	150 (22)	144 (19)	78 (16)	78 (17)
**del Zoppo 1998 [6]**	26	14	66.5 (11)	69.6 (11.1)	14:12	5:9	N/A	N/A	N/A	N/A	N/A	N/A

**Abbreviations**: **kg**: Kilogram; **mmHg**: Millimeters of Mercury; **N/A**: not applicable; **SD**: standard deviation.

## Data Availability

Data were publicly available.

## Supplementary Materials

The following supporting information can be downloaded at https://www.mdpi.com/article/10.3390/brainsci15050466/s1, Supplementary Figure S1. Shows the quality assessment of the RCT using ROB 2, Supplementary Figure S2. Shows leave one out analysis of NIHSS (0–1) rates at the end of follow-up, Supplementary Figure S3. Shows leave one out analysis of symptomatic ICH risk.
