# Supplementary material for: Efficacy and Safety of Recombinant Human Prourokinase in Acute Ischemic Stroke: A Systematic Review and Meta-Analysis of Randomized Controlled Trials
Source: Brain Sci. 2025 Apr 28;15(5):466. doi: 10.3390/brainsci15050466 (PMC12110422; doi:10.3390/brainsci15050466)

Supplementary Figure S1 [2-6,20]. Shows the quality assessment of the RCT using ROB 2

(a)

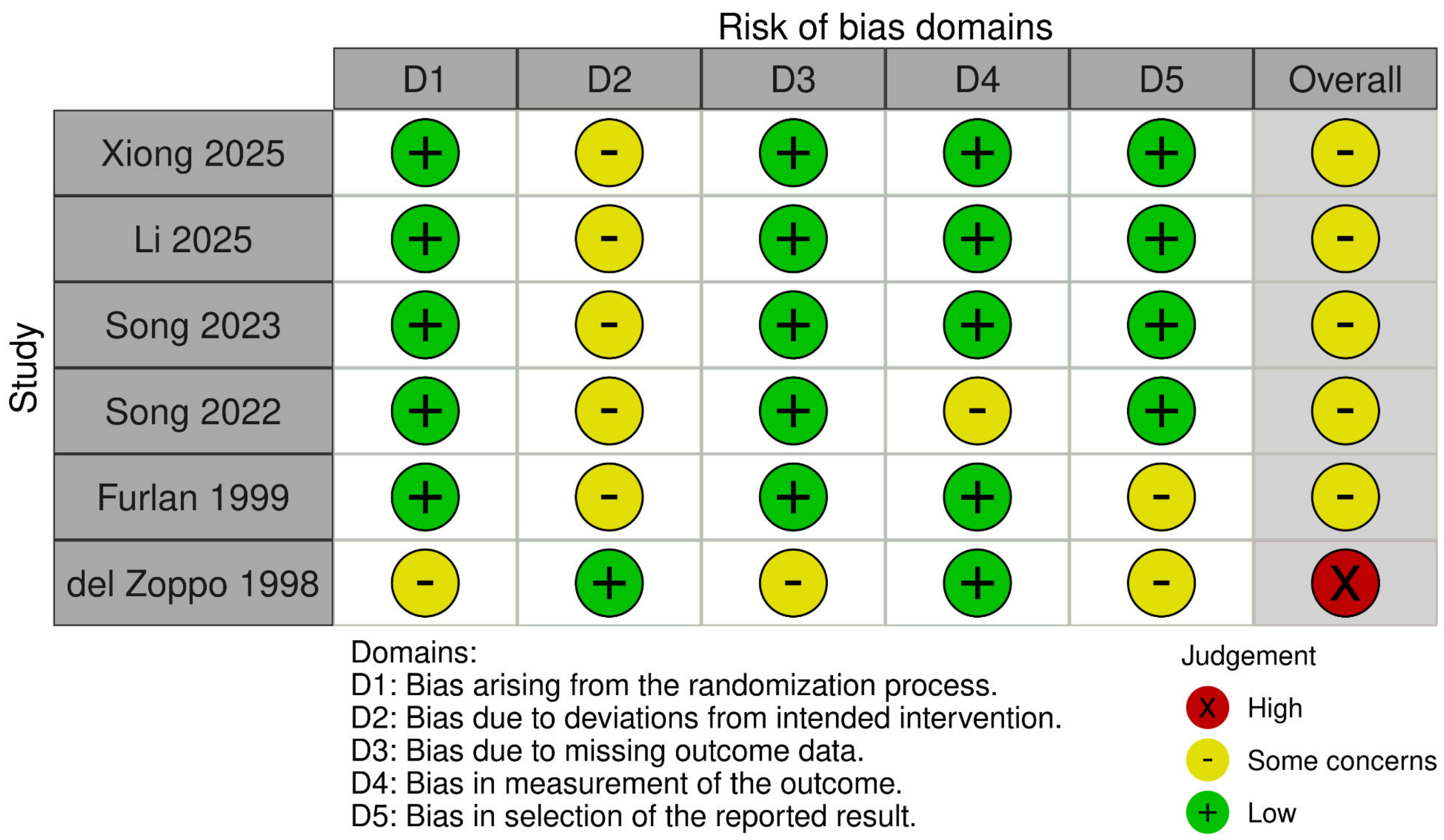

(b)

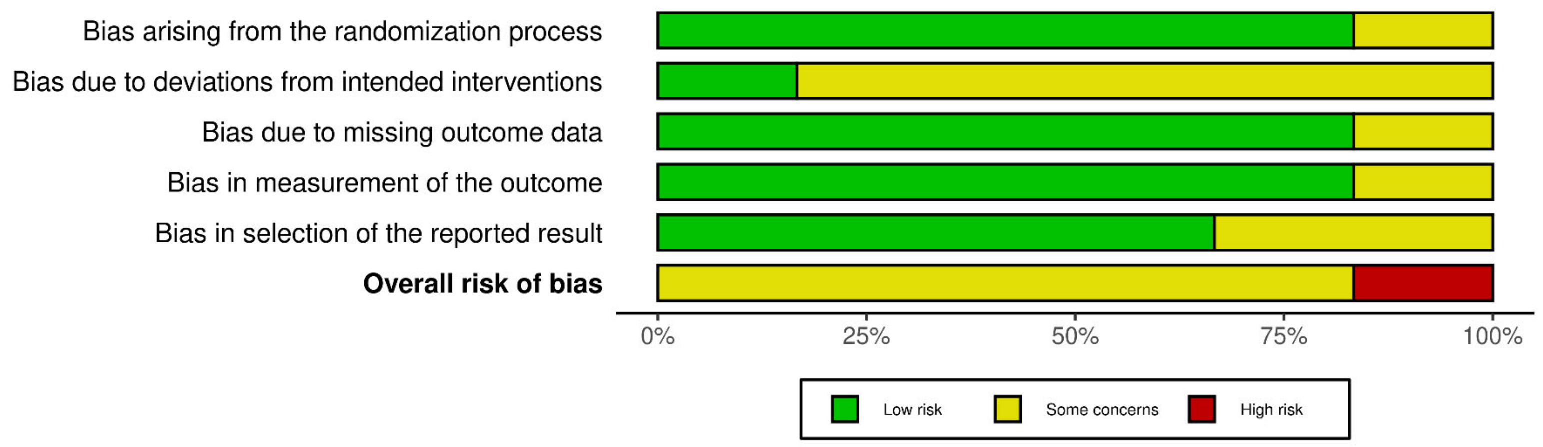

Supplementary Figure S2 [2-4]. Shows leave one out analysis of NIHSS (0-1) rates at the end of follow-up

Study or Subgroup	rhPro-UK Events	rhPro-UK Total	Comparator Events	Comparator Total	Weight	Risk Difference M-H, Random, 95% CI
**1.3.1 Intravenous recombinant human prourokinase (rhPro-UK), 35 mg vs. alteplase**						
Li 2025	538	775	540	777	70.4%	-0.00 [-0.05, 0.05]
Song 2022	13	36	11	38	3.3%	0.07 [-0.14, 0.28]
Song 2023	132	330	142	333	26.3%	-0.03 [-0.10, 0.05]
**Subtotal (95% CI)**		**1141**		**1148**	**100.0%**	**-0.01 [-0.04, 0.03]**
Total events	683		693			
Heterogeneity: Tau² = 0.00; Chi² = 0.84, df = 2 (P = 0.66); I² = 0%						
Test for overall effect: Z = 0.26 (P = 0.79)						

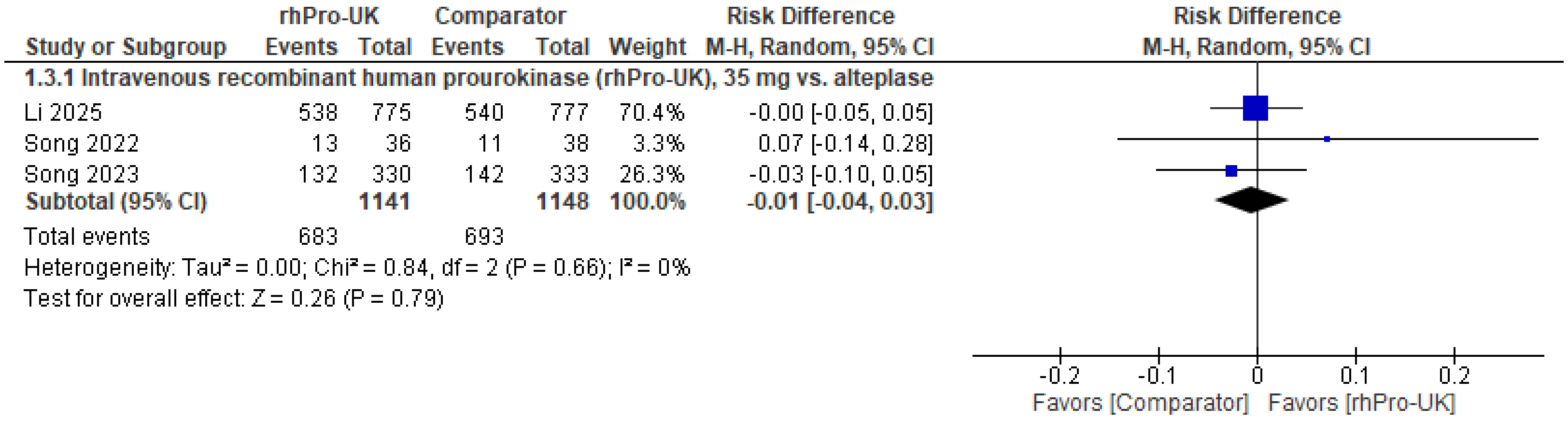

Supplementary Figure S3 [2-4]. Shows leave one out analysis of symptomatic ICH risk

Study or Subgroup	rhPro-UK Events	rhPro-UK Total	Comparator Events	Comparator Total	Weight	Risk Difference M-H, Random, 95% CI
**2.3.1 Intravenous recombinant human prourokinase (rhPro-UK), 35 mg vs. alteplase**						
Li 2025	2	770	10	775	81.3%	-0.01 [-0.02, -0.00]
Song 2022	0	36	0	38	2.4%	0.00 [-0.05, 0.05]
Song 2023	5	330	6	333	16.4%	-0.00 [-0.02, 0.02]
**Subtotal (95% CI)**		**1136**		**1146**	**100.0%**	**-0.01 [-0.02, -0.00]**
Total events	7		16			
Heterogeneity: Tau² = 0.00; Chi² = 0.65, df = 2 (P = 0.72); I² = 0%						
Test for overall effect: Z = 2.21 (P = 0.03)						

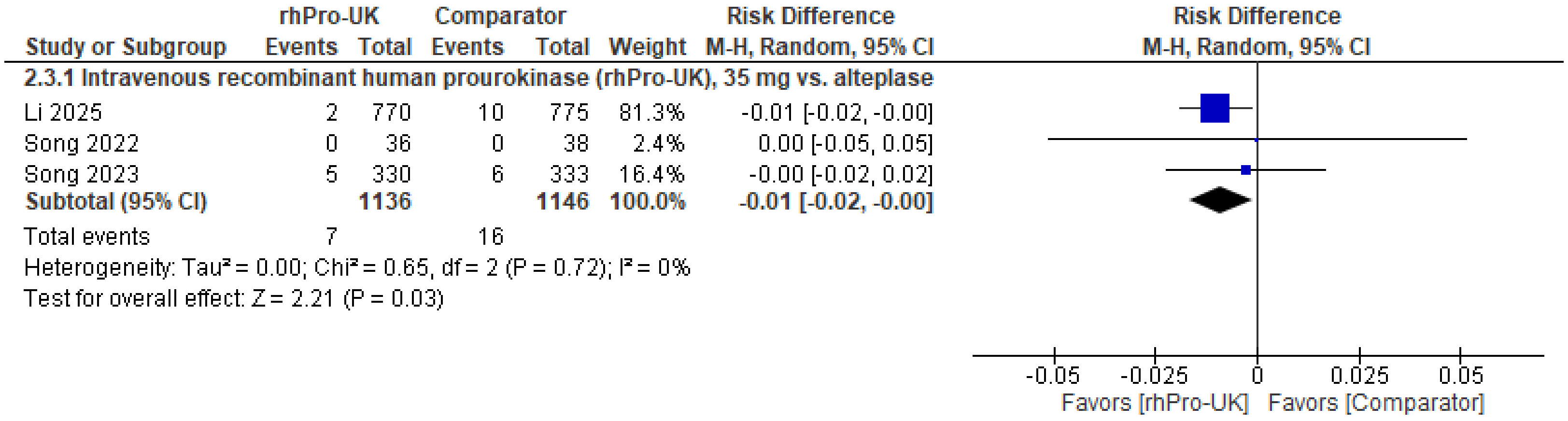

Supplement: Supplementary file 1 [file brainsci-15-00466-s001.zip › brainsci-3576091-supplementary.pdf]
